# Supplementary material for: Cryptic coral community composition across environmental gradients
Source: PLoS One. 2025 Feb 6;20(2):e0318653. doi: 10.1371/journal.pone.0318653 (PMC11801642; doi:10.1371/journal.pone.0318653)

a. Enterococcus

Mean:

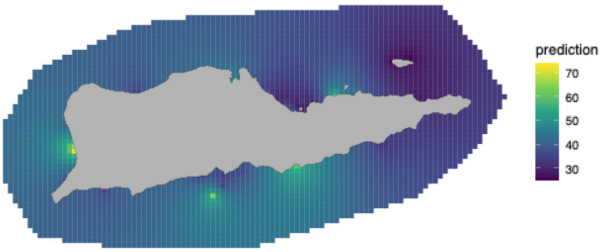

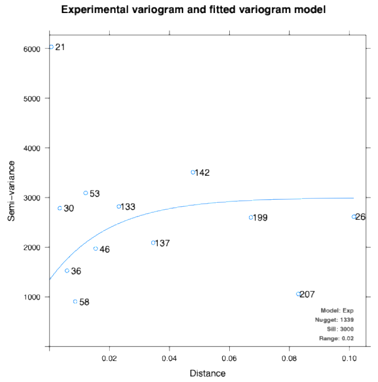

Maximum:

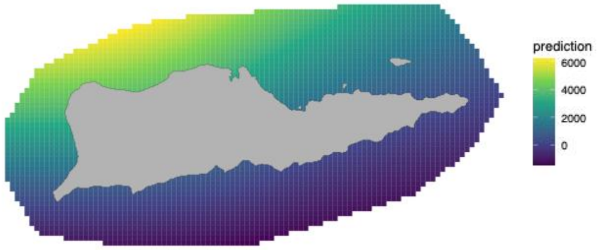

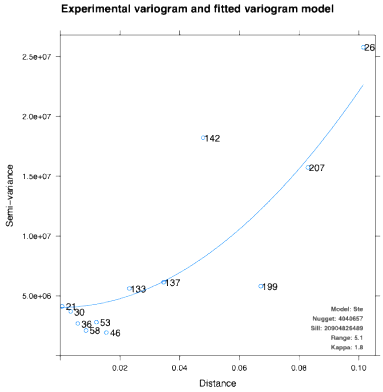

Monthly range:

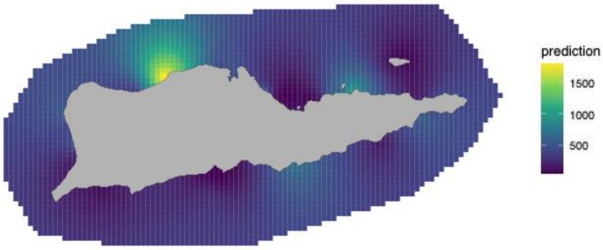

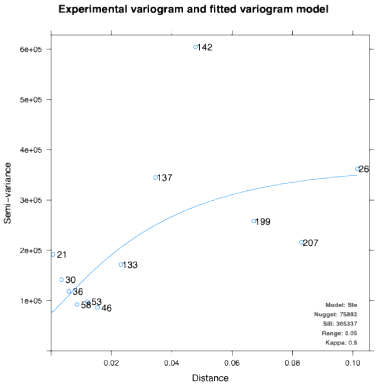

Yearly range:

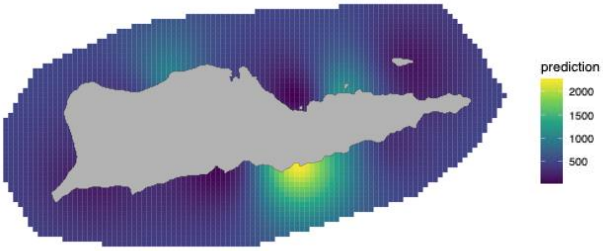

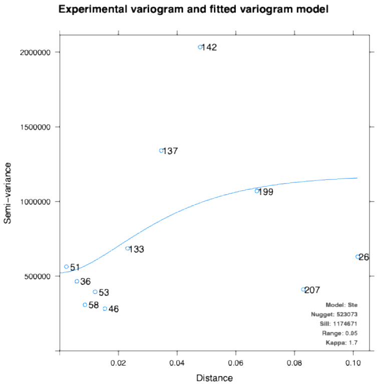

b. Dissolved oxygen

Mean:

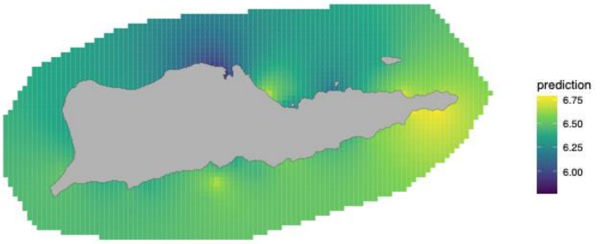

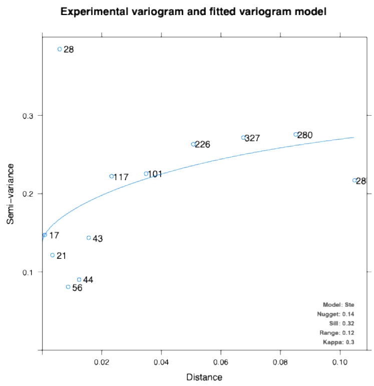

Maximum:

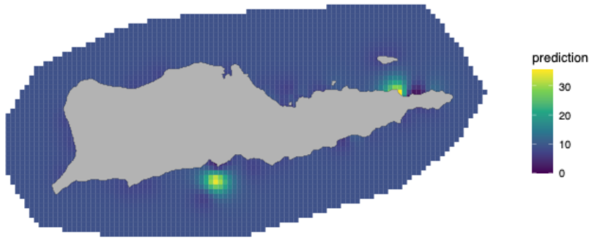

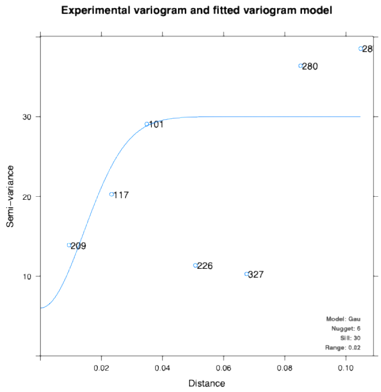

Minimum:

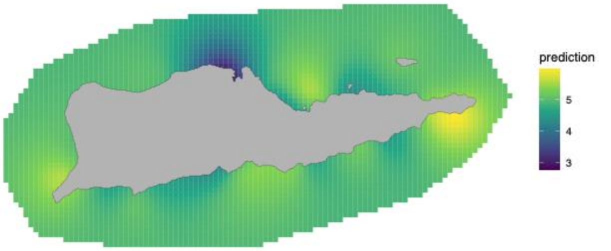

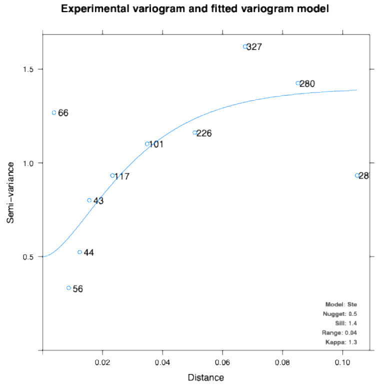

Monthly range:

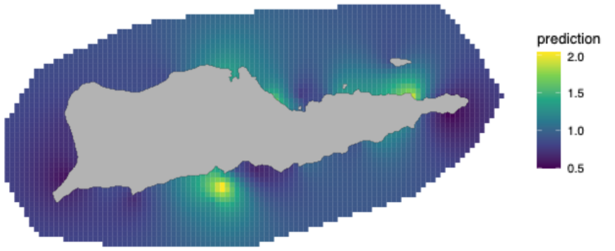

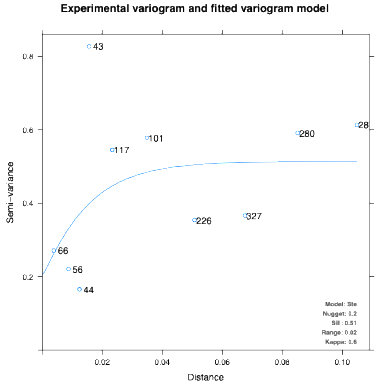

Yearly range:

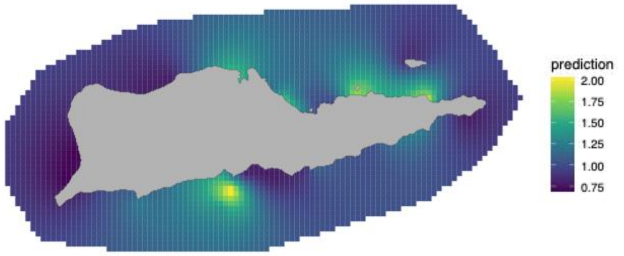

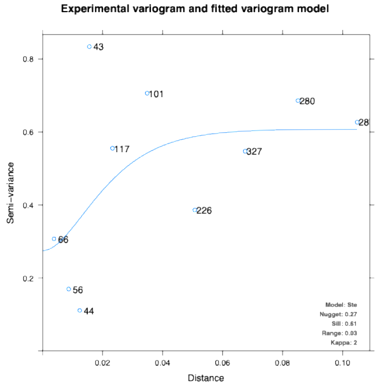

c. E. coli

Mean:

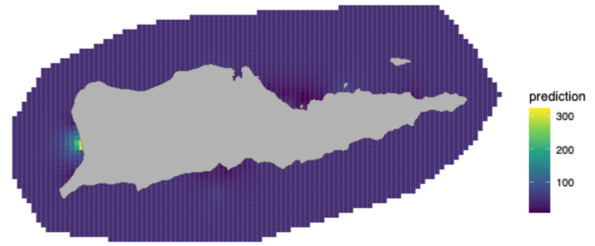

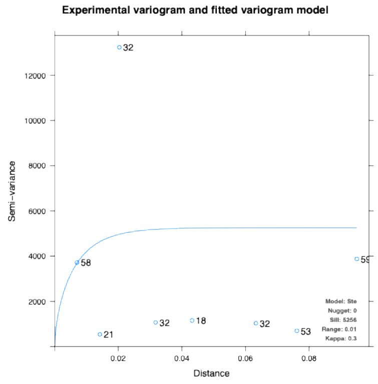

Maximum:

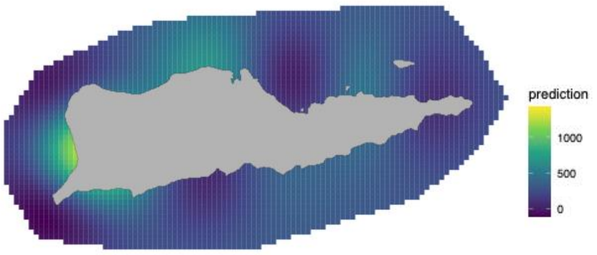

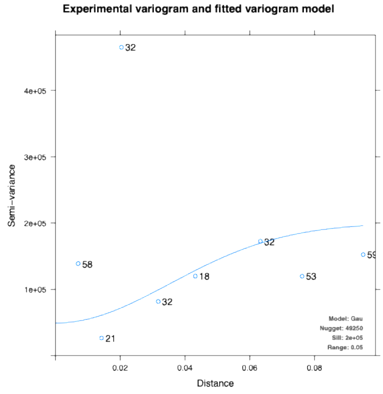

Monthly range:

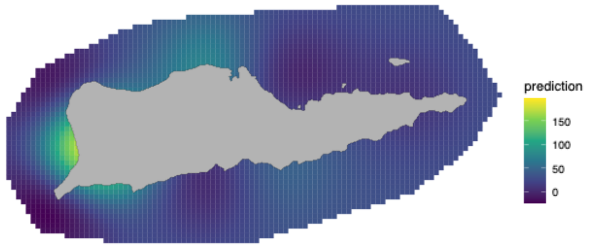

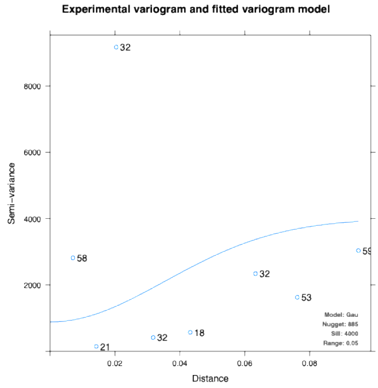

Yearly range:

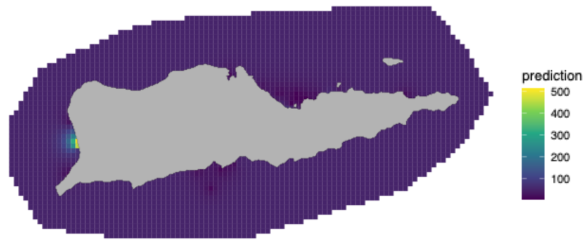

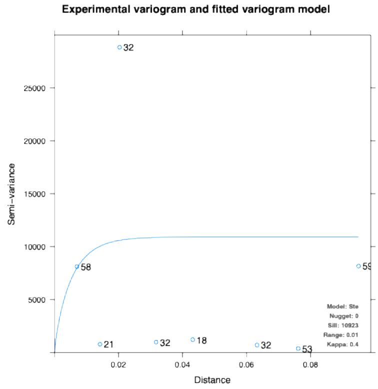

d. Nitrogen

Mean:

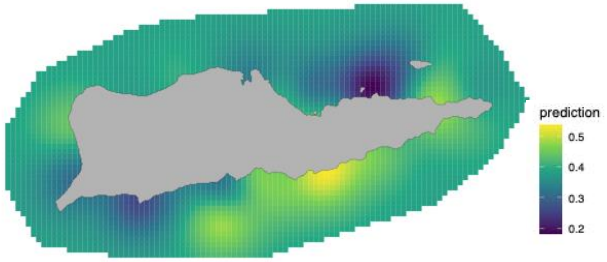

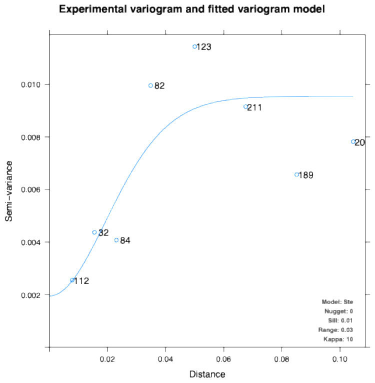

Maximum:

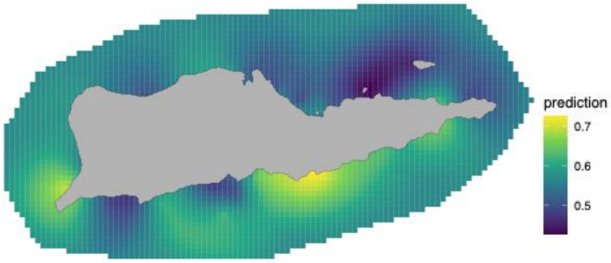

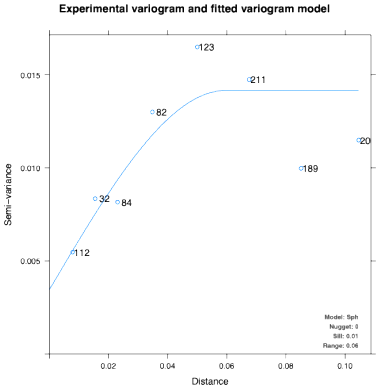

Monthly range:

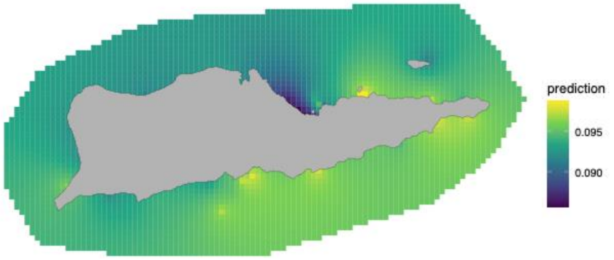

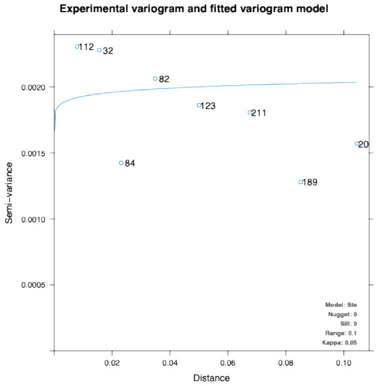

Yearly range:

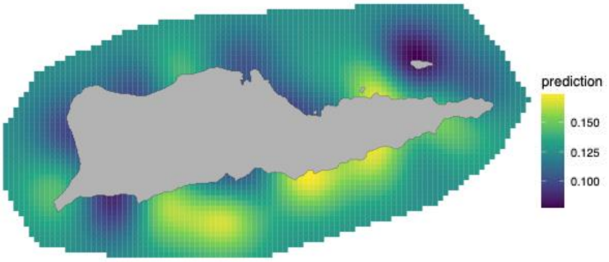

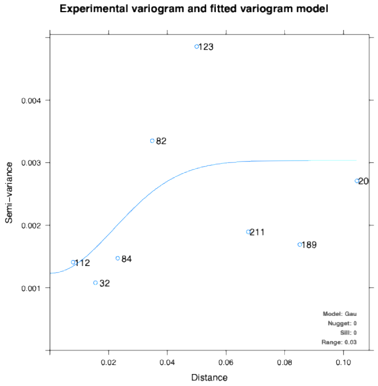

e. pH

Mean:

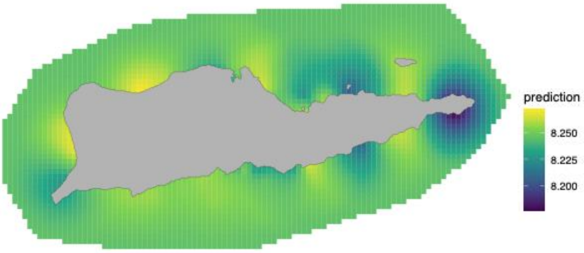

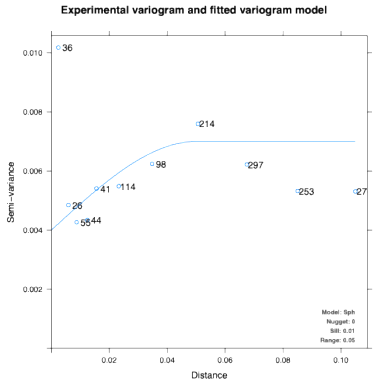

Maximum:

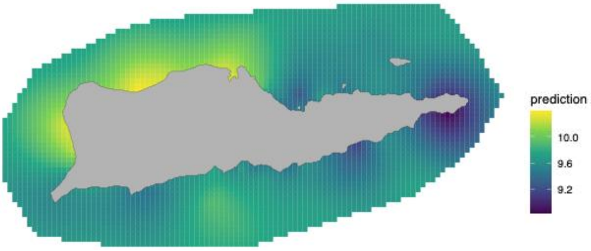

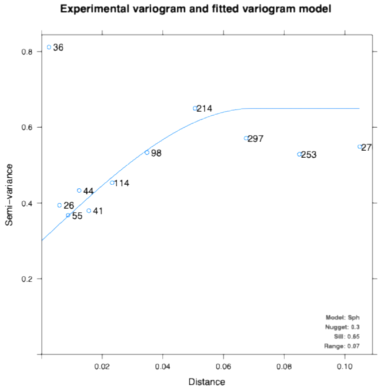

Minimum:

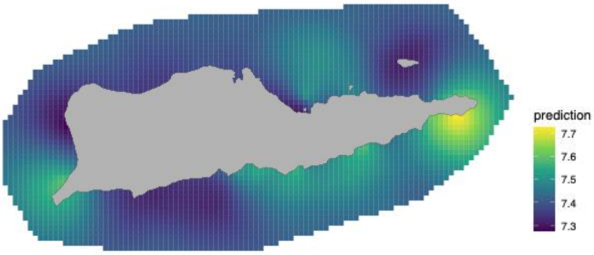

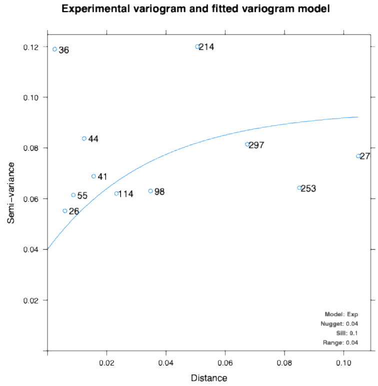

Monthly range:

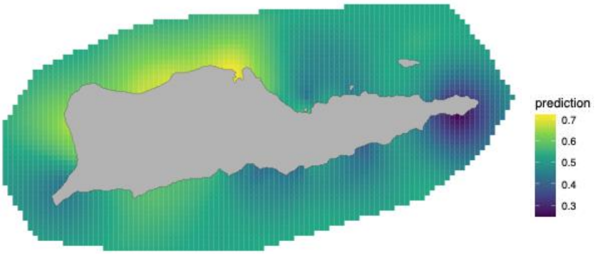

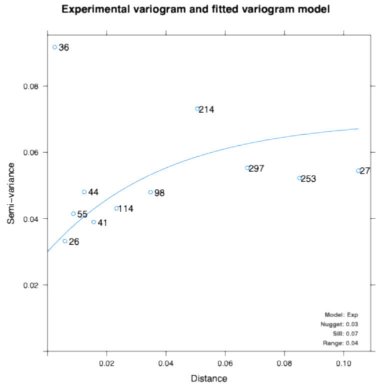

Yearly range:

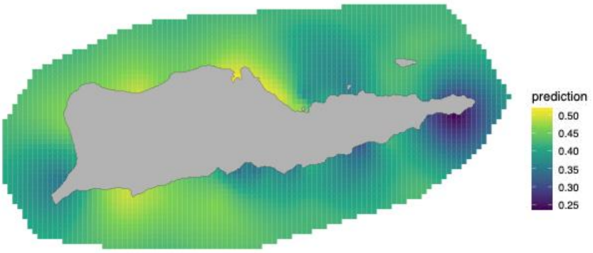

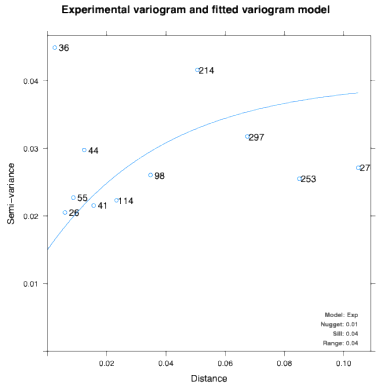

f. Phosphorus

Mean:

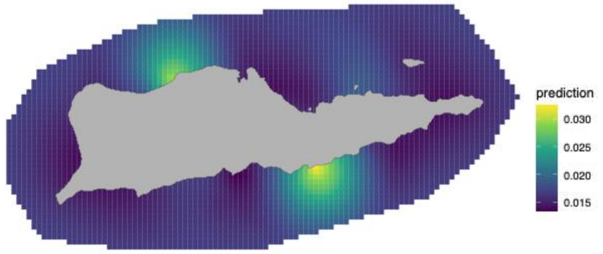

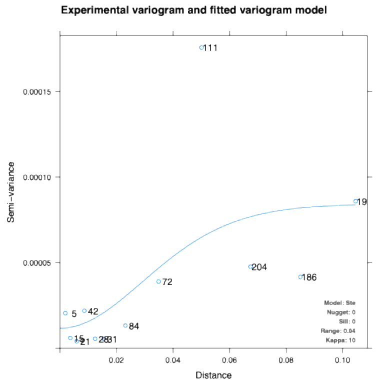

Maximum:

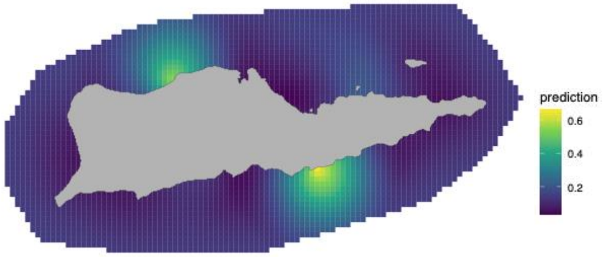

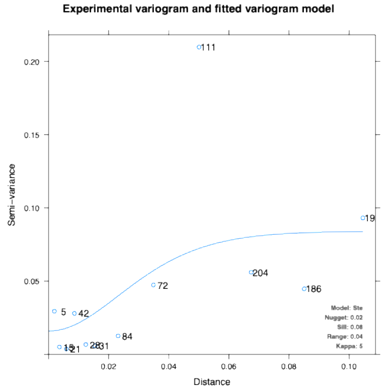

Minimum:

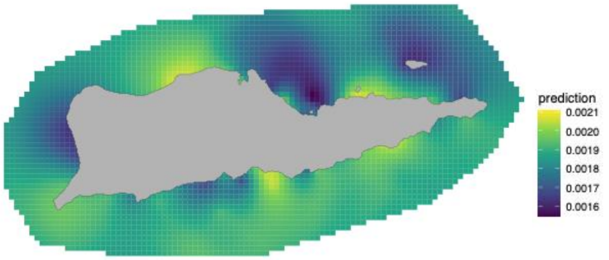

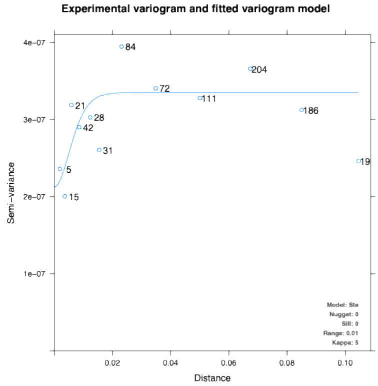

Monthly range:

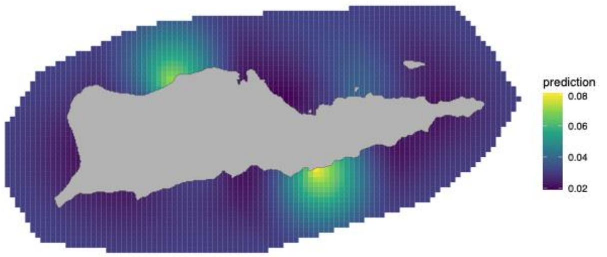

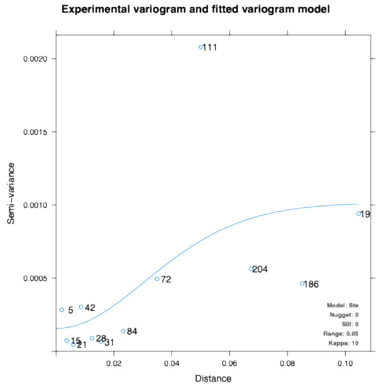

Yearly range:

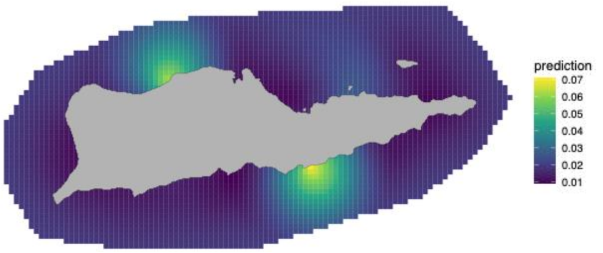

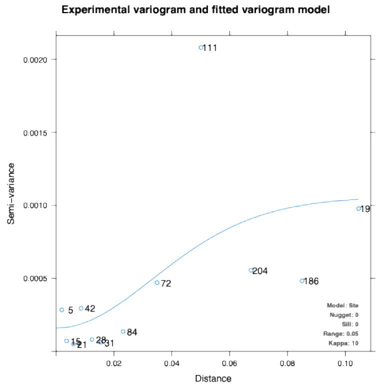

g. Secchi

Mean:

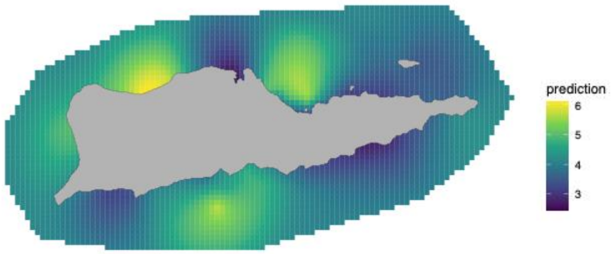

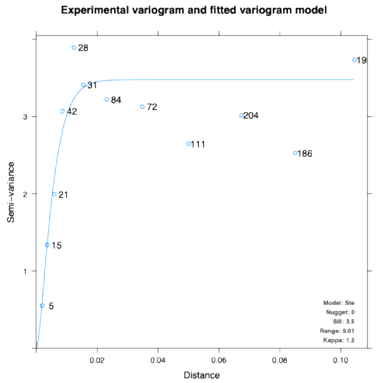

Maximum:

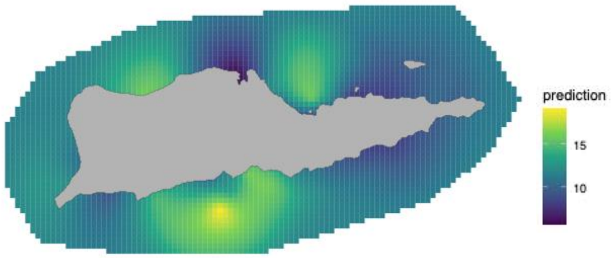

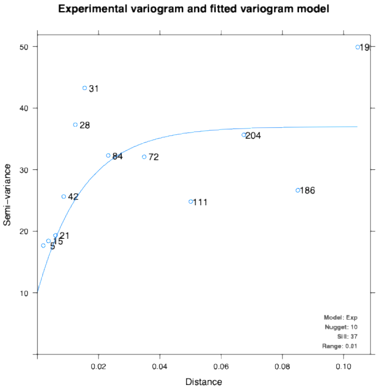

Minimum:

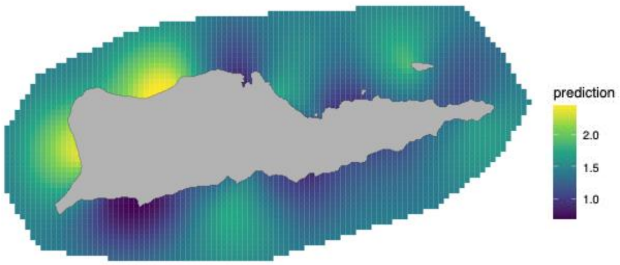

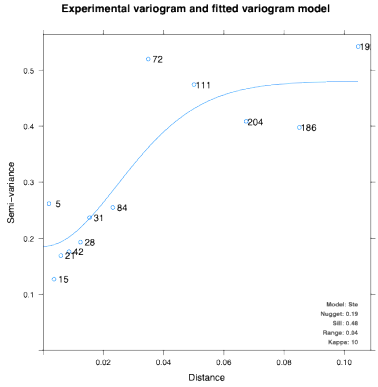

Monthly range:

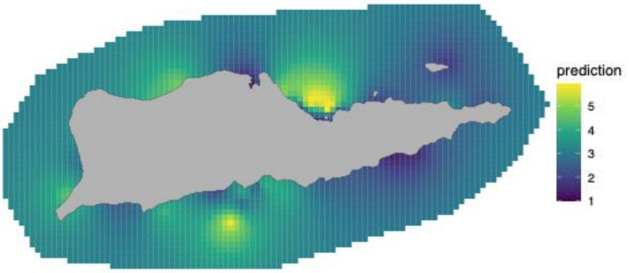

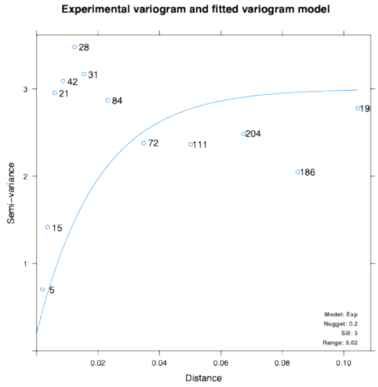

Yearly range:

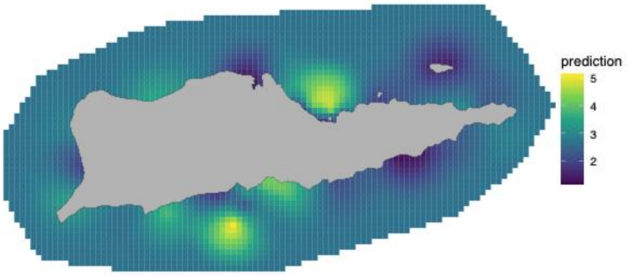

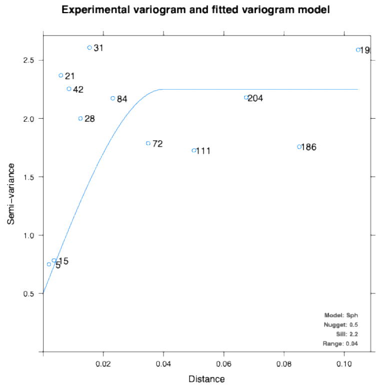

h. Temperature

Mean:

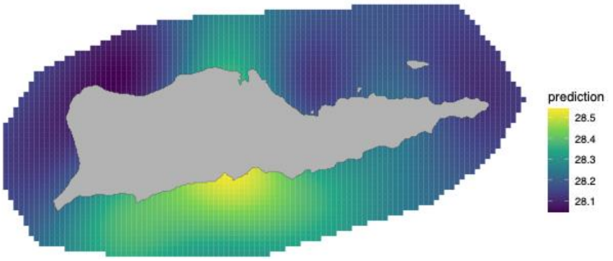

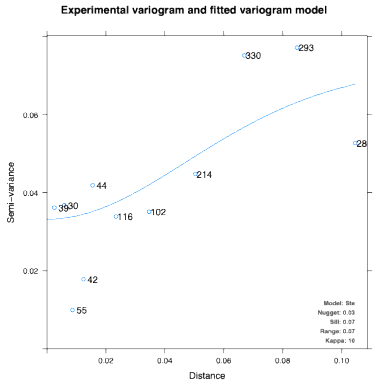

Maximum:

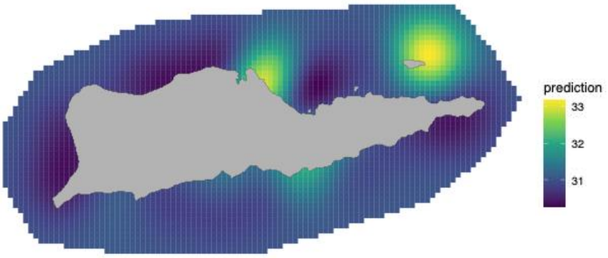

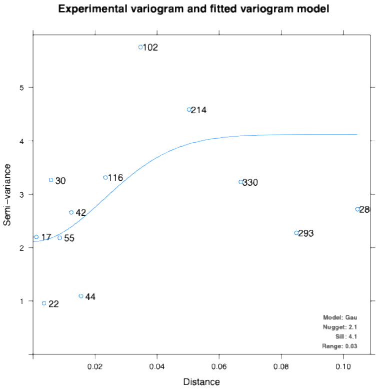

Minimum:

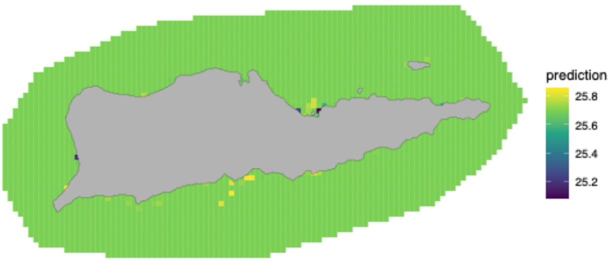

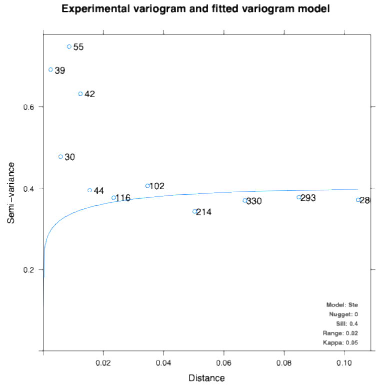

Monthly range:

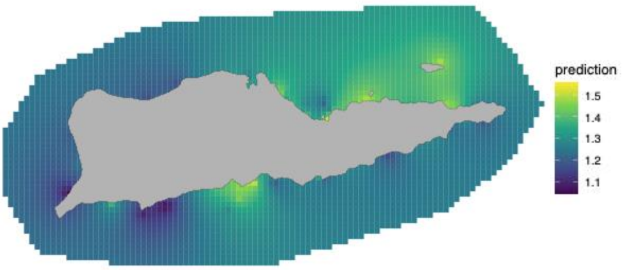

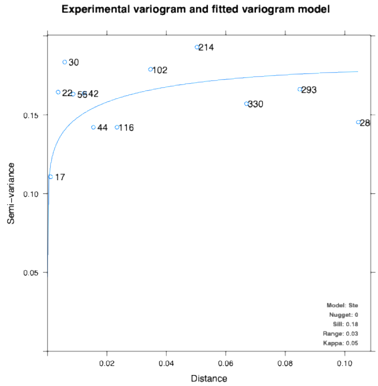

Yearly range:

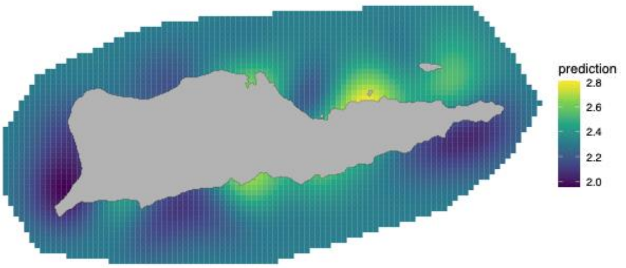

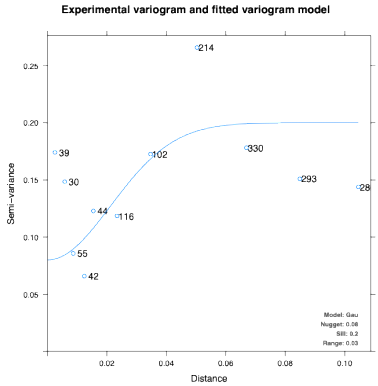

Supplement: S1 File — (ZIP) [file pone.0318653.s001.zip › SuppFig3.pdf]
