# Supplementary material for: Cryptic coral community composition across environmental gradients
Source: PLoS One. 2025 Feb 6;20(2):e0318653. doi: 10.1371/journal.pone.0318653 (PMC11801642; doi:10.1371/journal.pone.0318653)
Supplement: S1 File — (ZIP) [file pone.0318653.s001.zip › Supplemental_Material_revised2.docx]

**Supplemental Material**

**Supplementary section 1:** Genomic DNA isolation protocol

First, make the 2% CTAB extraction buffer:

Ingredients for 200 ml:

2 % CTAB a.k.a. Hexadecyltrimethylammonium bromide (4.0 g)

100 mM Tris pH 8 (20 ml of 1.0 M sol)

20 mM EDTA (16 ml of 0.25 M sol)

164 ml H2O

Dissolve CTAB before adding 1.4 M NaCl (16.4 g). Stir on a hot plate with a little warmth until CTAB is dissolved.

Then add NaCl and continue stirring on hot plate until dissolved.

The following amounts are for 1 sample. Before beginning: Get ice. Place aliquot of isopropanol in the freezer. Preheat heat block to 42°C. Preheat elution buffer aliquot to 65°C.

1. In a 2mL bead beater tube, add enough beads to fill the conical bottom of the tube. Then add **1.6µL** beta merceptoethanol, **1µL** proteinase K, and **1µL** RNAse A to **800µL** of CTAB extraction buffer. Place on ice.

2. Transfer coral fragment onto a clean kimwipe using clean forceps. Cut into smaller pieces with a clean razor blade if needed. Blot away excess ethanol.

3. Add sample to the tube with beads.

4.. Macerate sample in the bead beater for 40 seconds.

5. Incubate sample at 42°C for 1 hour or overnight.

6. Spin samples in a tabletop centrifuge for 15 minutes at max speed. Transfer aqueous phase to a clean gel-lock phase tube.

7. Add **800µL** (1 volume) chloroform/isoamyl alcohol (24:1) and vortex for a few seconds. Leave on ice 1 minute. Vortex again, 1-2 seconds.

8. Spin max speed for 20 minutes at 4°C.

9. Pipette off the aqueous phase and place in a clean 1.5 ml tube.

8. Add **550µL** (2/3 volume) of ice cold isopropanol and gently mix by inverting.

9. Incubate for 20 minutes at -20°C.

10. Centrifuge at max speed for 20 minutes at 4°C.

11. Pipette off the supernatant and discard.

12. Add **1000µL** 80% ethanol. Gently wash EtOH around the tube.

13. Centrifuge for 5 minutes at 4°C, max speed.

14. Pipette off the supernatant and discard. Let the pellets air dry upside down for 15 minutes in the hood.

15. Resuspend DNA in 30µL of warm (65°C) elution buffer.

16. Nanodrop and store samples at -20°C.

| **Variable** | **Years collected** | **# sites monitored** | **Minimum observations per site** | **Total observations** |
| --- | --- | --- | --- | --- |
| Enterococcus | 2011-2021 | 70 | 40 | 1,212 |
| Dissolved oxygen (DO) | 2011-2022 | 70 | 50 | 4,481 |
| Fecal Coliform (Ecoli) | 2000-2012 | 30 | 5 | 436 |
| Nitrogen | 2018-2022 | 55 | 10 | 967 |
| pH | 2012-2022 | 67 | 50 | 4,493 |
| Phosphorous | 2013-2022 | 55 | 30 | 1,932 |
| Secchi | 2013-2022 | 55 | 30 | 1,963 |
| Temperature | 2000-2022 | 70 | 50 | 8,734 |

**Supplementary Table 1:** Environmental variables monitored by the Virgin Islands Department of Planning and Natural Resources (DPNR). Note, however, that there was no environmental monitoring data from 2006-2007. These measurements were used to calculate mean, maximum, and minimum per site.

|  | Enterococcus | DO | Ecoli | Nitrogen | pH | Phosphorus | Secchi | Temp. |
| --- | --- | --- | --- | --- | --- | --- | --- | --- |
| Jan | 42 | 55 | - | 55 | 55 | 55 | 55 | 55 |
| Feb | 10 | 55 | 8 | 54 | 55 | 55 | 55 | 68 |
| Mar | 31 | 29 | 20 | - | 48 | 3 | 10 | 57 |
| Apr | 28 | 54 | 16 | - | 40 | - | - | 55 |
| May | 50 | 70 | 10 | 55 | 67 | 55 | 55 | 70 |
| Jun | 31 | 61 | 20 | - | 60 | 55 | 55 | 70 |
| July | 38 | 62 | 21 | - | 62 | 55 | 55 | 70 |
| Aug | 36 | 55 | 11 | 55 | 55 | 55 | 55 | 68 |
| Sep | 55 | 70 | 20 | - | 67 | 55 | 55 | 70 |
| Oct | 19 | 55 | 8 | 55 | 56 | 55 | 55 | 67 |
| Nov | 31 | 55 | 20 | 55 | 55 | 55 | 55 | 66 |
| Dec | 45 | 55 | 17 | - | 55 | 55 | 55 | 70 |

**Supplementary Table 2:** Number of environmental measurements for each variable per site in each month, used to calculate the average monthly range per site.

|  | Enterococcus | DO | Ecoli | Nitrogen | pH | Phosphorus | Secchi | Temp. |
| --- | --- | --- | --- | --- | --- | --- | --- | --- |
| 2000 | - | - | 13 | - | - | - | - | 53 |
| 2001 | - | - | 17 | - | - | - | - | 53 |
| 2002 | - | - | 14 | - | - | - | - | 53 |
| 2003 | - | - | 17 | - | - | - | - | 53 |
| 2004 | - | - | 27 | - | - | - | - | 53 |
| 2005 | - | - | 20 | - | - | - | - | 53 |
| 2008 | - | - | 13 | - | - | - | - | 55 |
| 2009 | - | - | 6 | - | - | - | - | 24 |
| 2010 | - | - | 11 | - | - | - | - | 54 |
| 2011 | 19 | 42 | 13 | - | - | - | - | 42 |
| 2012 | 54 | 55 | 19 | - | 52 | - | - | 55 |
| 2013 | 41 | 55 | - | - | 55 | 55 | 55 | 55 |
| 2014 | 48 | 55 | - | - | 55 | 55 | 55 | 55 |
| 2015 | 24 | 55 | - | - | 55 | 55 | 55 | 55 |
| 2016 | 43 | 55 | - | - | 67 | 55 | 55 | 70 |
| 2017 | 21 | 55 | - | - | 55 | 55 | 55 | 55 |
| 2018 | 44 | 55 | - | 55 | 55 | 55 | 55 | 55 |
| 2019 | 25 | 55 | - | 54 | 55 | 55 | 55 | 55 |
| 2020 | 17 | 55 | - | 55 | 55 | 55 | 55 | 55 |
| 2021 | 41 | 55 | - | 55 | 55 | 55 | 55 | 55 |
| 2022 | - | 55 | - | 54 | 55 | 54 | 55 | 55 |

**Supplementary Table 3:** Number of environmental measurements for each variable per site in each year, used to calculate the average yearly range per site.

|  | *A. agaricites* | *M. cavernosa* | *O.*  *faveolata* | *P. astreoides* | *P.*  *strigosa* | *S. siderea* |
| --- | --- | --- | --- | --- | --- | --- |
| Fredericksted Pier | 3 | 1 | 6 | 7 | 3 | 3 |
| Butler Bay | 8 | 9 | 9 | 9 | 13 | 2 |
| Carambola | 1 | 6 | 9 | 0 | 0 | 2 |
| North Star | 0 | 0 | 0 | 0 | 6 | 0 |
| Cane Bay | 1 | 14 | 10 | 14 | 4 | 5 |
| Columbus Landing | 3 | 0 | 0 | 0 | 0 | 0 |
| The Palms | 1 | 2 | 2 | 3 | 9 | 5 |
| WAPA | 0 | 0 | 5 | 0 | 3 | 0 |
| Deep End | 6 | 3 | 4 | 7 | 17 | 3 |
| Llew’s Reef | 0 | 0 | 0 | 0 | 5 | 0 |
| Channel Rock | 0 | 0 | 0 | 0 | 4 | 0 |
| Isaac Bay | 2 | 0 | 2 | 7 | 6 | 1 |

**Supplementary Table 4:** Sample sizes per species per location, retained after sequencing and filtering. Sites are listed directionally so that top to bottom rows correspond with west to east. Sites (rows) are also colored by ecoregion, in accordance with Fig. 2. At some sites, only a few species were sampled- such as *A. agaricites* at Columbus Landing or *P. strigosa* at Llew’s Reef and Channel Rock- either due to limited species present at that site or limited sampling efforts. Despite differential representation across sites, most species have representation across ecoregions.

**Supplementary Figure 1:** Sampling locations and depth distributions (in meters) for each coral species. *A. agaricites*, *P. astreoides*, and *P. strigosa* are mostly found at shallow sites, while the other species can be found at 30 meters and beyond.

**Supplementary Figure 2:** Sites along the St. Croix coastline monitored from 2009-2022 by the Virgin Islands Department of Planning and Natural Resources (DPNR), representing eight *in situ* variables.

**Supplementary Figure 3 (see separate attachment):** Predictions of each environmental variable across the map of St. Croix, as inferred via kriging interpolation of environmental monitoring data.

Semi-variograms are included to the right of each map, which depict spatial autocorrelation between environmental measurements across the seascape. The x-axis of the variogram plot refers to the “lag distance,” or the distance between pairs of data points (expressed here in degrees of latitude and longitude). Data points are grouped into bins based on their lag distance, so that all pairs of points with similar distances are analyzed together. Thus, each point in the variogram plot represents the average semivariance for all data pairs within a bin. By default, the R package *automap* ensures that each bin contains at least five data pairs; if any bin has fewer than five pairs, it is merged with the adjacent bin. The y-axis represents the calculated semi-variance at each distance bin. Finally, the label next to each point indicates the number of data pairs used to calculate the semi-variance at each distance bin.

From the resulting variogram plots, several parameters controlling the fit of the model can be interpreted- including the “nugget,” or the y-intercept of the variogram, which represents small-scale variability of the data likely attributed to measurement error. The “range” is the distance where the variogram levels off (i.e., distance where spatial autocorrelation reduces), and the “sill” is the variance where the variogram levels off.

When interpreting the variogram, lag distance increases along the x-axis. For certain variables, such as mean temperature, the semivariance on the y-axis generally increases, indicating a reduction in spatial correlation between data points as they become further apart. For other variables, such as minimum temperature, semivariance appears relatively constant across lag distances, implying a more uniform spatial distribution of values through space.

Regarding the fit of the variogram model, we note that the initial points in the plot often exhibit high variability due to the small lag distances between data pairs. These points are calculated from fewer data pairs, as the number of available pairs tends to increase with larger distances. Moreover, the high variability among the initial points is often an artifact of observations being sparse or clustered around certain regions.

For example, in our study, the high variability at small lag distances in all variables is most likely due to more frequent sampling near the capital, Christiansted (see Supplementary Fig. 1), where environmental monitoring efforts are more concentrated. These localized measurements contribute disproportionately to the small lag distance bins. To minimize the impact of this artifact, we fit the variogram model to the remaining data points, excluding the early, high-variability points.

Some variogram models show a better fit than others (ex. mean nitrogen, mean dissolved oxygen, and mean temperature), thus the resulting interpolations for those variables are more likely to capture actual trends across the seascape. On the other hand, models with a poorer fit can still identify regions with exceptionally high values (ex. mean E. coli) or low values (ex. monthly range of Nitrogen). However, we opted to retain even these gradients as predictors to see if those spatial patterns correlated with genetic structure. If variables with weaker variograms demonstrated high associations with genetic structure, we would have interpreted that result as genetic turnover across the seascape without attributing it to our interpolated variables, as even well-predicted variables would need to be validated experimentally. We did not, however, retain variables without variability across the seascape (ex. minimum nitrogen) and removed those variables as predictors from gradient forest models.
