# Supplementary material for: Cryptic coral community composition across environmental gradients
Source: PLoS One. 2025 Feb 6;20(2):e0318653. doi: 10.1371/journal.pone.0318653 (PMC11801642; doi:10.1371/journal.pone.0318653)
Supplement: Attachment — Submitted filename: STX_ReviewerResponse (1).pdf [file pone.0318653.s002.pdf]

Journal Requirements:

When submitting your revision, we need you to address these additional requirements.

1. Please ensure that your manuscript meets PLOS ONE's style requirements, including those for file naming.

> >>>**Response** – We verified that our submission, supporting file names, and figure file names meet the PLOS ONE style requirements, per the submission guidelines.

2. Thank you for stating the following financial disclosure: This study was funded by The Nature Net Fellowship from The Nature Conservancy to K.L.B.. Please state what role the funders took in the study. If the funders had no role, please state: ""The funders had no role in study design, data collection and analysis, decision to publish, or preparation of the manuscript."" If this statement is not correct you must amend it as needed. Please include this amended Role of Funder statement in your cover letter; we will change the online submission form on your behalf.

> >>>**Response** – The above statement is correct, and we added it to the cover letter. We also removed our funding statement from the acknowledgements, per the PLOS ONE submission guidelines.

3. We note that Figures 1 and 2 in your submission contain [map/satellite] images which may be copyrighted. All PLOS content is published under the Creative Commons Attribution License (CC BY 4.0), which means that the manuscript, images, and Supporting Information files will be freely available online, and any third party is permitted to access, download, copy, distribute, and use these materials in any way, even commercially, with proper attribution. For these reasons, we cannot publish previously copyrighted maps or satellite images created using proprietary data, such as Google software (Google Maps, Street View, and Earth). For more information, see our copyright guidelines: http://journals.plos.org/plosone/s/licenses-and-copyright.

We require you to either (1) present written permission from the copyright holder to publish these figures specifically under the CC BY 4.0 license, or (2) remove the figures from your submission.

> >>>**Response** – The shoreline in our figures comes from the Global Self-consistent, Hierarchical, High-resolution Geography Database (GSHHG, https://www.soest.hawaii.edu/pwessel/gshhg/), which is released under the GNU Lesser General Public License. We added a citation to this resource in the Data Availability section of the manuscript. The photos in Figure 1 are original, taken by the field team in

St. Croix during sample collection for this project, and not published or uploaded anywhere else.

Reviewer Comments to Author:

Reviewer #1:
General comments
Evaluating how population and community structure is influenced by environmental conditions is key to understanding the influence of environmental change on ecosystems and implementing effective restoration/conservation efforts. This study explores the genetic structure of coral communities around St. Croix and how that genetic structure relates to a spatiotemporal variation in environmental conditions. The authors find variable genetic structuring across coral taxa with certain environmental conditions of key influence on coral community structure over space and time.

Generally, I found this manuscript to be well-written, well-structured and easy to read. Moreover, the quantitative design appears to be well constructed and performed. However, I feel that the manuscript would benefit from greater detail on portions of the analysis and environmental data structure (expanded on below). My technical understanding of these genetic methods is limited and thus I cannot provide meaningful technical review of this section.

Although the study is designed to be a more exploratory assessment, an important consideration here is the lack of specific hypotheses that are set out to be tested. While this isn't necessarily a make-or-break issue, it is important for how the study is framed. The authors suggest that the broader implication of this work is around conservation and restoration actions—understanding where corals perform well and poorly can inform where corals are out planted. However, I find the interpretation of results provided here to be a bit of a stretch in a restoration context. Simply put, it is difficult to assess whether corals perform better or worse in a particular area without causal-type experimentation and hypothesis testing, indeed the authors indicate that such examination is a logical next step. This association-type analysis evaluates how spatial variation in genetic structure of corals is associated with long-term trends in environmental conditions, but it doesn't say how these variables actually affect corals or their genetic structure. Given that consideration, reframing portions of the introduction and discussion to be focused on how environmental conditions can contribute to genetic heterogeneity might be a better framing than trying to make the leap regarding restoration efficacy.

>>>**Response** – We thank the reviewer for a thorough review of our study. We agree that a formal hypothesis presented in the introduction would improve the framing of our

manuscript. Due to the large number of studies identifying depth as a possible driver of cryptic divergence and the growing concern about temperature anomalies imposing selective pressure on coral reefs, we hypothesize that depth and temperature may shape cryptic coral community composition. After evaluating the literature surrounding prior conjecture related to depth and temperature, we decided to add this hypothesis to the introduction:

L85-87: "Given the consistent associations of depth and temperature with cryptic genetic variation in coral species, we hypothesize that these factors may serve as key environmental drivers of cryptic coral community composition."

Secondly, we agree with the reviewer that our findings improve our understanding of how spatial variation in genetic structure is associated with long-term environmental conditions, with minimal implications about restoration efficacy. However, coral restoration is a major theme in the Caribbean, and these types of studies lay the groundwork to test causal relationships between environmental variables and coral genotypes. To more accurately discuss the implications of this study, we rephrased much of our manuscript to investigate community-environment associations, but we retained some references to restoration implications in the first two introductory paragraphs for vital context of our study. Then we suggest future studies that could aid restoration efforts in the last sentences of our discussion and conclusion.

Specific comments:
Introduction

L53-55: "ecologically cohesive communities". What is an ecologically cohesive community? Does this imply some type of facilitation by heterospecifics? This is not defined here or in the referenced literature. The potential facilitation effect may not be entirely relevant to this study.

>>>**Response –** "Ecologically cohesive" was redundant phrasing meant to describe the community, and we did not intend to imply any facilitation by heterospecifics. We agree with the reviewer that this term is confusing and adds little to this statement, so we removed the term to shorten the sentence.

L51-52: "These species likely form communities that are locally adapted to specific, yet undefined, ecoregions."

L60: The authors note species and genera. Better to note these as coral "taxa" not species.

>>>**Response –** Fixed.

L58: "Many coral taxa around the world, including Acropora hyacinthus…"

L64: The referenced literature, Grupstra et al. (2024 in NEE), reviews the potential drivers of genetic divergence among cryptic corals. I wouldn't necessarily call this divergence for unknown reasons as the authors purport in this line. Rather it appears that there are many supported hypotheses for why provided by Grupstra et al. and by the authors in the following sentence. This context is a critical component of this study given that the authors do not generate specific hypotheses regarding why certain environmental attributes influence genetic structure. Perhaps a more useful approach would be to evaluate the hypotheses, pre or post-hoc, generated by Grupstra et al. within the study system, species.

>>>**Response** – This is true, Grupstra et al. provides many potential hypotheses that would be applicable to our study. We selected depth and temperature as possible variables that influence cryptic coral community structure, and we added a hypothesis to our introduction. We also revisit this hypothesis in the discussion, to show how our findings aligned with our initial expectations. We believe that this addition of a hypothesis provides important context that greatly improves our manuscript.

L85-87: "Given the consistent associations of depth and temperature with cryptic genetic variation in coral species, we hypothesize that these factors may serve as key environmental drivers of cryptic coral community composition."

L443-444: "…our findings align with our hypotheses that depth and temperature are critical environmental drivers influencing genetic divergence in cryptic communities…"

L77-79: I appreciate the broadening of this topic to relevant examples in temperate ecosystems. However, I would suggest focusing this section on the abiotic factors that influence marine communities exclusively. Perhaps the broader strokes would be more useful earlier in the introduction.

>>>**Response –** This is a good suggestion. We removed the terrestrial examples from this sentence and added more marine examples to focus on abiotic factors that affect marine communities.

L73-75: "In addition to depth, environmental factors such as salinity (Dupont et al., 2014; Pecuchet et al., 2016) and temperature (Abreu et al., 2023; Flanagan et al., 2018) significantly influence community composition in marine ecosystems."

L89-91: "highlighting the intricate relationship between genetic variation and environmental challenges in coral ecosystems." Can the authors be more specific regarding environmental challenges? Are they referring to climate change?

>>>Response – By "environmental challenges" we were simply referring to naturally occurring selective pressures, but we can see why this wording caused confusion. We removed this sentence and rephrased the paragraph to discuss temperature impacts on coral populations.

L73-84: " In addition to depth, environmental factors such as salinity (Dupont et al., 2014; Pecuchet et al., 2016) and temperature (Abreu et al., 2023; Flanagan et al., 2018) significantly influence community composition in marine ecosystems. Temperature is of particular concern due to increasing heat anomalies leading to mass mortality events (Hoegh-Guldberg et al., 2007; Muñiz-Castillo et al., 2019; Pandolfi et al., 2011). Some cryptic lineages exhibit variable responses to temperature (Rose et al., 2021; Gómez-Corrales & Prada, 2020). For example, cryptic lineages of O. faveolata in Panama show different physiological responses to coral bleaching, suggesting adaptations to rising water temperatures (Gómez-Corrales & Prada, 2020). Likewise, cryptic lineages of A. hyacinthus in American Samoa demonstrate variations in heat tolerance and their association with heat-resistant symbionts (Rose et al., 2021). More broadly, shifts in community composition can result from divergent responses to heat stress (Hughes et al., 2018), although communities may recover if environmental conditions reverse (Gouezo et al., 2019)."

L93-104: This paragraph seems like it should be in the methods section. The authors note potential mechanistic drivers of these patterns in the previous paragraph. Perhaps this space could be used to introduce specific hypotheses that the authors are testing?

>>>Response – We agree that we needed a hypothesis in our paper, so we added our hypothesis to the previous paragraph. However, we believe this paragraph should stay in the introduction as it only provides an overview of our methods. This overview is important due to the many existing methodologies available, and we are simply providing context for why we opted to implement gradient forest models over other methods.

Moreover, this paragraph does not contain any information that would be important for reproducing our methods- all of that is provided in the actual methods section.

L89-100: "A common framework for finding associations between community composition and environmental variables is to compare species abundance at multiple sampling sites across a heterogeneous landscape with various environmental factors (Anderson & Willis, 2003). Several methods are used for this analysis, including latent factor mixed models (LFMM; Frichot et al., 2013), redundancy analysis (RDA; Forrester et al., 2013), and gradient forest (Ellis et al., 2012). Gradient forest may be the most advanced method, as it employs multiple regression trees to estimate environmental thresholds that drive community turnover across the landscape. This method, which extends the principles of random forest to handle multiple response variables, allows for the detection of both linear and nonlinear relationships between communities and their environments, while also controlling for collinearity among environmental variables (Ellis et al., 2012). In our study, we apply the gradient forest method to identify the environmental predictors of coral community composition across the reefs of St. Croix in the U.S. Virgin Islands."

Methods

L153-155: This passage would benefit from additional detail regarding sampling design. What was the size distribution per species of colonies sampled? Are these similarly sized colonies? What is the sample size per species per site?

>>>Response – We did not measure colony size during sample collection, so we cannot provide this data. But generally, adult colonies >5cm were sampled at all sites. We added the following phrase to reflect this:

L151-152: "Adult colonies >5cm in diameter were sampled, and for each sample, depth was recorded in situ (Supplementary Fig. 1)."

L222-223: Can the authors expand on the structure and processing of environmental data? Mainly, what was the temporal resolution of environmental data used and subsequently summarized? Perhaps this information can be added to Supplementary Table 1. Moreover, interpretation of the environmental heterogeneity across sites and years would benefit from an additional supplementary figure illustrating spatiotemporal variation in each environmental variable.

>>>**Response –** We added **new Supplementary Tables 2 and 3**, which shows the temporal extent of environmental monitoring. Supplementary Table 2 shows the number of observations per year for each environmental variable, and these observations were used to summarize the average yearly range at each site. Supplementary Table 3 shows the number of observations per month for each variable, which were used to summarize the average monthly range at each site. To illustrate the spatial extent of environmental monitoring, we also added **new Supplementary Fig. 1** to show the location of sites where environmental variables were measured.

We also thank the reviewer for suggesting that we go back to check the temporal resolution of environmental data, because we realized that some of our calculations of mean monthly range and mean yearly range included months or years with only a few observations that could skew the whole interpolation. In order to produce Supplementary Tables 2 & 3, we cleaned up our environmental monitoring data by removing months or years that included spurious observations. Then we re-interpolated each variable (now in Supplementary Fig. 2), re-extracted values at coral sampling sites, and re-inferred ecoregions (**new Fig. 2**). The only difference this created in our results is that Columbus Landing was previously in Ecoregion D, but now it appears to be part of Ecoregion B. This re-categorization makes more logical sense, given the location of Columbus Landing and the alignment of clusters with an east-west distribution.

We also reran both gradient forest models with the redefined environmental predictors. For the model with ecoregions, this resulted in depth outperforming ecoregions in R^2 weighted importance (**new Fig. 5a**). For the model with all variables, depth remained the top predictor, but maximum temperature became the second most important predictor (**new Fig. 5d**). We changed Fig. 5c and f as well, to reflect these outcomes.

L227: Kriging interpolation appears to be an appropriate way to interpolate environmental conditions for the location of colonies sampled. However, evaluating interpolation performance is contingent on the location of monitoring sites relative to colony locations. Where were the environmental monitoring sites? How far apart were they? Can the authors provide a supplementary figure of the variograms generated from kriging interpolation?

>>>**Response –** We added **new Supplementary Fig. 1** to show the location of monitoring sites used for interpolation. Most sites occur near the capital Christiansted; however, sites are well-distributed across the entire coastline of St. Croix. We also added **new Supplementary Fig. 2** to show the variograms and predictions generated for each variable.

L235-236: The authors note that the "optimal number of genetic lineages was visually determined" (L206). Was a similar method performed for the environmental data (i.e., ecoregions and environmental data)? For ecoregions, what was the visual criteria for ecoregion grouping? Was there consideration of the height of each site similarity?

>>>**Response** – Yes, a similar method was performed for determining the ecoregions from environmental data. For the ecoregions, the visual criteria for ecoregion grouping was based on the first three partitions in the hierarchical clustering tree (Fig. 2b). There are differences in the height of each site similarity (for instance, sites in Ecoregion C are more similar to each other than those in Ecoregion B), however, the resulting groups of four ecoregions are well-supported by the natural east-to-west progression along the coastline.

L239-252: "The first three partitions of the hierarchical clustering tree resulted in four ecoregions (Fig. 2c) across the seascape. At first glance, these regions appear to align with visual differences between the reef environments (Fig. 1). These regions are also reasonable given the influence of the Caribbean Current around St. Croix. The northbound Caribbean current deflects around the southern shore of St. Croix, sending disparate wake flows to the eastern sites (ecoregion A) and the western sites (ecoregions C and D) (Chérubin & Garavelli, 2016). The two flows are asymmetric due to wake eddies, or circular currents, forming contained benthic conditions in ecoregion A (Chérubin & Garavelli, 2016). Ecoregion B (The Palms and WAPA) are closest to the Virgin Islands capital of Christiansted, and likely receive land-based sources of pollution (Oliver et al., 2011). Ecoregion C (Cane Bay, North Star, and Carambola) is designated within ecoregion D, but likely differs due a prominent shelf break that drops 5,500m deep approximately 250m from shore (Hubbard et al., 2008). Together, these ecoregions likely capture broad environmental heterogeneity across the seascape, so that even variables that are missing from this analysis are likely congruent with these environmental boundaries."

L260: It appears that two gradient forest models were employed here: one that assessed the influence of the 40 environmental predictors and another that assessed the effect of depth and the four ecoregions. Is this interpretation correct? This section would benefit for an explicit statement regarding the model structure[s].

>>>**Response** – Yes, that is correct. We added clarification here that two models were employed:

L267-268: "We ran two gradient forest models: one with depth and assignment to four ecoregions one with all 40 environmental predictors."

Results

Table 1: What were the sample sizes per species per location? Are the sample sizes adequate to characterize genetic variation within and between locations? Additionally, in Fig. 4, there is some indication that genetic diversity appears to increase with sample size (panel b., M. cavernosa) although these patterns may not be as evident for the other 5 taxa. It is possible that the diversity of genetic linkages revealed may be an artifact of sampling design, although it is challenging to assess this without explicit reporting of sampling size per site per species. Do sample sizes reflect the relative abundance of each taxa at each site?

>>>**Response –** Question by question:

1. What were the sample sizes per species per location?
    a. We added **new Supplementary Table 4**, which shows the sample sizes per species per location.
2. Are the sample sizes adequate to characterize genetic variation within and between locations?
    a. Sample sizes varied across sites, however sample sizes are distributed well across the ecoregions. We acknowledge that *M. cavernosa*'s sample size may be small, compared to the number of lineages detected. However, since we were able to detect representatives of all six known Caribbean lineages, we doubt that we missed any lineages in our sampling effort.
3. Additionally, in Fig. 4, there is some indication that genetic diversity appears to increase with sample size (panel b., *M. cavernosa*) although these patterns may not be as evident for the other 5 taxa. It is possible that the diversity of genetic linkages revealed may be an artifact of sampling design, although it is challenging to assess this without explicit reporting of sampling size per site per species.
    a. The high genetic diversity in *M. cavernosa* in Fig. 4 is due to the high genetic diversity of *M. cavernosa* across the Caribbean. We agree with the reviewer that our *M. cavernosa* samples would not be sufficient to delineate six lineages on their own. Thus, we aligned our samples with samples from previous studies of *M. cavernosa* populations from Florida, Cuba, and Curacao (see lines pasted below). Thus, the diversity of *M. cavernosa* cannot be an artifact of sampling design.

        L297-300: " The M. cavernosa and S. siderea sample sets also clustered with samples from prior studies (Gallery et al., 2023; Rippe et al., 2021;

Sturm et al., 2023). M. cavernosa from St. Croix clustered with six lineages and S. siderea clustered with three lineages from Florida and the Gulf of Mexico."

4. Do sample sizes reflect the relative abundance of each taxa at each site?
    a. Sample sizes reflect relative abundance at some sites- for instance Deep End has an extraordinary amount of *P. strigosa* compared to other species, so *P. strigosa* is highly represented at Deep End. However, we aimed to sample all species at each site as much as possible.

L338: The authors refer to Fig. 1d here. Should this be Fig. 2c? Its difficult to tell.

>>>**Response –** Thank you for catching this, it has been changed to Fig. 2c.

L334: The amount of variation in coral community structure explained by environmental conditions in the two separate models appears to be relatively small (12.5 and 18.9%), with cross validation values quite low for environmental predictors? Could this be a result of overfit models (i.e., too many environmental predictors)? Can the authors provide detail as to whether hierarchical cluster models and subsequently gradient forest models were assessed with fewer environmental variables? A more parsimonious environmental cluster model could contribute to increased gradient forest model performance. It might be beneficial to provide an explanation of why the variance explained was relatively low (e.g., low sample size).

>>>**Response –** Question by question:

1. The amount of variation in coral community structure explained by environmental conditions in the two separate models appears to be relatively small (12.5 and 18.9%), with cross validation values quite low for environmental predictors? Could this be a result of overfit models (i.e., too many environmental predictors)?
    a. Reducing the number of predictors is not necessary for gradient forest models because they can account for inflation of predictor importance among correlated predictors using conditional permutation. Rather, it is optimal (and recommended) to retain all predictors in gradient forest (Ellis et al. 2012).
2. Can the authors provide detail as to whether hierarchical cluster models and subsequently gradient forest models were assessed with fewer environmental variables? A more parsimonious environmental cluster model could contribute to increased gradient forest model performance.

a. Hierarchical cluster models *were* assessed with fewer environmental variables (see Fig. 2a). For gradient forest models, we illustrate our point above by implementing gradient forest with fewer predictors (i.e., with the subset reduced for multicollinearity in Fig. 2a). The resulting amount of variation explained is approximately the same (see figure pasted below), thus reducing the number of predictors does not increase gradient forest model performance.

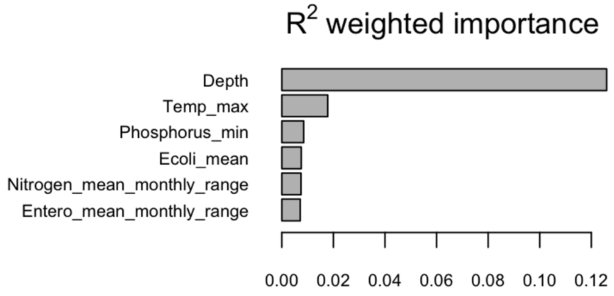

b. It might be beneficial to provide an explanation of why the variance explained was relatively low (e.g., low sample size).
   i. Genetic variance explained by the environment is typically low in natural systems (ex. Bay et al. 2018), since there are many non-environmental factors that influence population structure which are unaccounted for- such as ocean currents, reproductive timing, anthropogenic influence, etc. In this study, depth is actually a remarkably strong predictor of genetic variation. An additional explanation is that R-squared values from gradient forest are based on cross-validation and are always lower than R-squared from classical linear regression, computed on the same data as used for building the model (and therefore inflated due to overfitting).

L346: The authors refer to depth being the strongest predictor of community composition. However, Fig. 5a shows Ecoregion to be the most important, followed by depth. Do they mean Fig. 5b?

> **>>>Response –** Yes, we had meant to say Fig. 5a. However, after rerunning both gradient forest models with improved predictors (see response to Lines 222-223 for Reviewer 1), depth becomes the strongest predictor for both models. We reflected this change in the text:
>
> *L339-342: "In the first gradient forest model, depth and ecoregions together accounted for 15.4% of the variation in cryptic coral communities. Depth was the strongest predictor (cross-validation R^2 = 0.083) and Ecoregion B, representing central St. Croix*

near the capital Christiansted (Fig. 2c), was the most important ecoregion driving community structure (Fig. 5a)."

Figure 4: Add figure legend for bubble size corresponding to sample size.

>>>Response – Done!

Figure 5: Remove titles from panels A and B and provide x-axis labels.

>>>Response – Done!

Discussion

L376: The authors note that community composition is partially shaped by environmental conditions, mainly ecoregions and depth. Indeed, there is a high degree of variation in coral community structure that isn't explained by models. While the authors expand on other potential drivers of these dynamics (e.g., cyclones temperature anomalies etc.), it is important to note that the temporal resolution of environmental conditions is not matched to the instantaneous measure of genetic structure. In other words, the authors assess how the environmental legacy of sites over ~12 years contributes to "current" community genetic structure. This approach makes many assumptions about what the coral community 'looked like' over this 12-year period. An acknowledgement and brief discussion of this assumption is probably needed here.

>>>Response – This is a great point by the reviewer. We added an acknowledgement about this:

L381-387: "However, we note that these associations reflect how community structure correlates with long-term environmental trends- in this case, abiotic variables summarized over 9-22 years. Further investigations into instantaneous selective pressures, such as cyclones or bleaching events, could be conducted by resampling coral over time. Comparing abundance before and after such events could provide insights into how cryptic communities shift in response to these acute pressures, thereby enhancing our temporal understanding of coral responses to changing environmental conditions."

Reviewer #2:

Comments to the Author:
This is a nice study and a well-written paper. Strengths are that the distribution of cryptic coral lineages were studied in many (six) sympatric species from many (12) sites. One potential weakness might be that the number of individuals sampled per species was perhaps a bit low in some species (n = 21, 25 spread across 8 sites) so potential that some composition-environment patterns might not be fully characterized (but this is hard to know).

The manuscript is presented well, and it was easy to read. The data analyses all seem robust to me. I think the result will be useful to those working at St. Croix as well as to the bolster the evidence that cryptic species of corals are becoming the rule rather than the exception.

The overall result is consistent with previous studies that many morphologically-defined species contain evolutionarily distinct genetic lineages, that these lineages commonly co-occur but differ in relative abundance across depths and locations that differ in environmental variables. This study finds a correlation with yearly pH variation.

My only main comments are:
1) Because the association between community composition and environmental variables is still a correlation (and this is acknowledged e.g., 459), the discussion could perhaps be strengthened by considering alternative explanations for the current composition-distribution pattern that can be made without invoking environmental variables, like dispersal patterns / limitation or that they are just a signature of past disturbance history.

> >>>**Response –** We added some alternative explanations (i.e., dispersal patterns) to our discussion of community spatial patterns:
>
> *L450-460: "Untangling the mechanisms driving and maintaining genetic differentiation between cryptic lineages in the absence of geographic isolation poses challenges. Previous theories regarding sympatry and parapatry have proposed various mechanisms for this phenomenon, such as stepped clines driving tension zone formation and assortative mating (Bolnick & Fitzpatrick, 2007; Carneiro et al., 2013; Singhal & Moritz, 2012; Prada & Hellberg, 2021). Additionally, the observed spatial patterns may not solely result from environmental heterogeneity across the seascape, but may also result from other factors, such as ocean currents influencing larval dispersal (Cherubin et al. 2016). However, we suspect that the formation of cryptic benthic communities may rely on post-recruitment selection, given the continuity of seascape gradients and the ability of currents to sustain high dispersal and connectivity."*

2) Genetic lineages within species were identified by visual examination only (of the hierarchical clustering tree and PCoA). The clustering analyses included samples from prior studies, which increases the confidence in the visual determination of lineages. However, the evidence for how the current samples cluster with previous samples is hard to find. It might be good to show which tips in Figure 3 are the "reference" samples from previous studies, and make a better connection to the nomenclature of each lineage (if a consistent one even exists for these species?), so readers can trace what lineages are showing up where.

>>>**Response –** We are unable to paste figures including the reference samples into our main or supplemental documents because they are dissertation chapters currently in prep or in revision for publication elsewhere. However, we *can* paste those figures here in the response for the reviewers.

For *A. agaricites*, we clustered the St. Croix samples with a larger dataset (n=250) from the Florida Keys. This dataset comes from a dissertation chapter (Black, 2024) and a preprint (Black et al. 2022, in revision), which is included in the cited literature. In the PCA below, the yellow, blue, and indigo clusters represent cryptic genetic lineages that are shallow-specialist, shallow-preferred, and depth-tolerant, respectively. Red samples represent those from this study in St. Croix, and they cluster with the blue and indigo lineages from Florida.

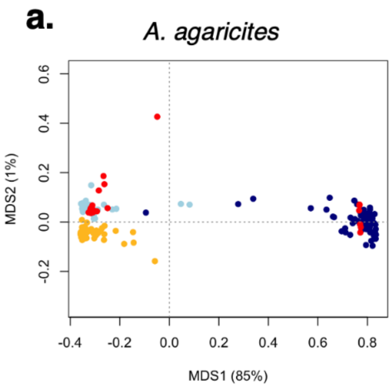

For *P. astreoides*, we also clustered St. Croix samples with a larger dataset (n=299) from the Florida Keys. These data were also part of the prior referenced study (Black, 2024; Black et al. 2022, in revision). Similar to the above species, the yellow, blue, and indigo clusters represent shallow-specialist, shallow-preferred, and depth-tolerant lineages. Grey samples represent hybrids between these three lineages, and red and black samples denote the two lineages found in St. Croix. When evaluating MDS1 and MDS2, we see that the St. Croix samples appear closest to the yellow and blue lineages, though most St. Croix samples are in the positive range of MDS1, and both MDS1 and MDS2 only depict a small range of genetic variation. When evaluating MDS1 and MDS3, the St. Croix

samples appear in their own quadrant that separates them from Florida samples. Together, these PCAs suggest that St. Croix samples are separate lineages from those found in Florida. When evaluating MDS2 and MDS6, the PCA is oriented in the same way as in the referenced study (without St. Croix samples included), and the St. Croix samples appear closest to the yellow and grey samples from the Florida Keys.

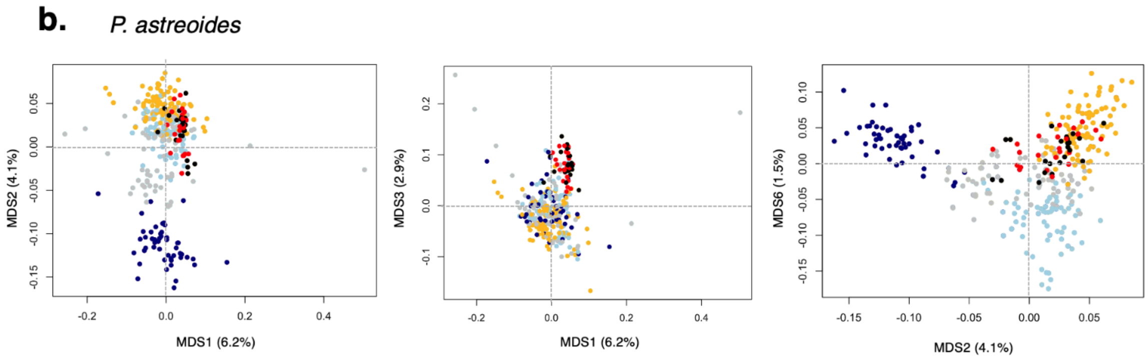

For *M. cavernosa*, we clustered the St. Croix samples with a larger *M. cavernosa* dataset from across the Caribbean- including samples from Florida, Cuba, and Curacao. This figure comes from a dissertation chapter (Gallery, 2024) and a manuscript in preparation (Gallery et al. 2024, in prep.). *M. cavernosa* has six lineages repeatedly found throughout the Caribbean (also previously reported in Rippe et al. 2021 and Sturm et al. 2023), which are also specialized by depth (lineage names- i.e., MC4, MC7, etc.- refer to the average depth where each lineage is found) (Gallery, 2024). Samples from St. Croix cluster well with each of these lineages.

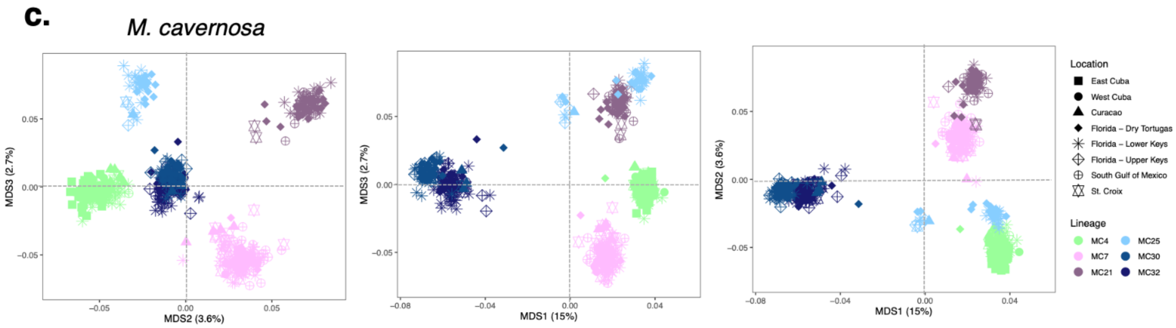

For *S. siderea*, we clustered the St. Croix samples with a larger dataset from Florida, previously published in Rippe et al. 2021 (n=128). In the PCA below, the gold and yellow clusters refer to two shallow-distributed lineages, while the dark blue and purple clusters refer to two deep-distributed lineages. The gray samples are hybrids between lineages, and the St. Croix samples are in red.

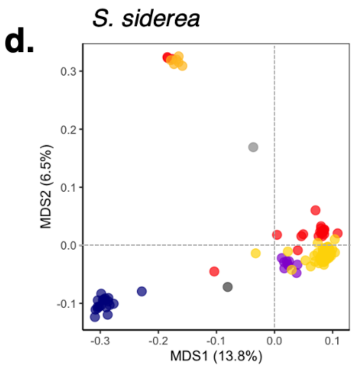

Minor:

Line 224: Could you mention here what the frequency of recording are, and what the mean, maximum, etc refer to (mean per day, per month? How many measurements comprised each mean?). This is just to get a better idea of how well the environmental variables are characterized.

> >>>**Response** – We added **new Supplementary Tables 2 and 3** to show the frequency of recording environmental variables. Supplementary Table 2 shows the number of observations per site each year, which was used to calculate the average yearly range per site. Supplementary Table 3 shows the number of observations per site each month, which was used to calculate the average monthly range per site. We also added the following text to clarify our calculations of mean, maximum, and minimum:
>
> *L222-226: "We summarized variables at each location by calculating mean, maximum, and minimum values across all observations. We also calculated mean monthly range as the difference between the maximum and minimum value at each site each month, and then averaged across months (Supplementary Table 3). Similarly, we calculated mean yearly range as range of values at each site recorded over each year, averaged across years."*

Line 227: Again, could you mention the spatial resolution of the sampling (how many sites?). Just trying to understand the robustness of the spatial interpolations. If a site where coral is sampled is far from the nearest site where environmental variables were measured, how good are the environmental estimates?

> >>>**Response** – We added **new Supplementary Fig. 1** to show the spatial resolution of environmental observations. Generally, environmental variables were monitored across

all coastlines of St. Croix, and very close to coral sampling sites. The only exception is that E. coli was not monitored near Cane Bay on the north shore.
