# Supplementary material for: Cryptic coral community composition across environmental gradients
Source: PLoS One. 2025 Feb 6;20(2):e0318653. doi: 10.1371/journal.pone.0318653 (PMC11801642; doi:10.1371/journal.pone.0318653)
Supplement: Attachment — Submitted filename: STX_ReviewerResponse_v2.docx [file pone.0318653.s003.docx]

**Journal Requirements:**

Please review your reference list to ensure that it is complete and correct. If you have cited papers that have been retracted, please include the rationale for doing so in the manuscript text, or remove these references and replace them with relevant current references. Any changes to the reference list should be mentioned in the rebuttal letter that accompanies your revised manuscript. If you need to cite a retracted article, indicate the article’s retracted status in the References list and also include a citation and full reference for the retraction notice.

**>>>Response –** The following references were removed because they too broadly supported our discussion of alternative explanations for community structure:

1. Bolnick DI, Fitzpatrick BM. Sympatric Speciation: Models and Empirical Evidence. Annu Rev Ecol Evol Syst. 2007 Dec 1;38(Volume 38, 2007):459–87.
2. Carneiro M, Baird SJE, Afonso S, Ramirez E, Tarroso P, Teotónio H, et al. Steep clines within a highly permeable genome across a hybrid zone between two subspecies of the European rabbit. Mol Ecol. 2013;22(9):2511–25.
3. Kolodziej G, Studivan MS, Gleason ACR, Langdon C, Enochs IC, Manzello DP. Impacts of Stony Coral Tissue Loss Disease (SCTLD) on Coral Community Structure at an Inshore Patch Reef of the Upper Florida Keys Using Photomosaics. Front Mar Sci. 2021 Aug 6;8.
4. Singhal S, Moritz C. Strong Selection Against Hybrids Maintains A Narrow Contact Zone Between Morphologically Cryptic Lineages In A Rainforest Lizard. Evolution. 2012 May 1;66(5):1474–89.

The following references were added to more specifically support alternative explanations for community structure- i.e., ocean currents, reproductive strategies (e.g., brooding vs. spawning), natural disturbances, and prezygotic barriers- as recommended by Reviewer 2.

1. Dai, C.-F., Fan, T.-Y., & Yu, J.-K. (2000). Reproductive isolation and genetic differentiation of a scleractinian coral Mycedium elephantotus. *Marine Ecology Progress Series*, *201*, 179–187. https://doi.org/10.3354/meps201179
2. Dunnavant, J. P., Flewellen, A. O., Jones, A., Odewale, A., & White, W. (2018). Assessing Heritage Resources in St. Croix Post‐Hurricanes Irma and Maria. *Transforming Anthropology*, *26*(2), 157–172. https://doi.org/10.1111/traa.12130
3. Fogarty, N. D., Lowenberg, M., Ojima, M. N., Knowlton, N., & Levitan, D. R. (2012). Asymmetric conspecific sperm precedence in relation to spawning times in the Montastraea annularis species complex (Cnidaria: Scleractinia). *Journal of Evolutionary Biology*, *25*(12), 2481–2488. https://doi.org/10.1111/j.1420-9101.2012.02625.x
4. Gilmour, J. P., Underwood, J. N., Howells, E. J., Gates, E., & Heyward, A. J. (2016). Biannual Spawning and Temporal Reproductive Isolation in Acropora Corals. *PLOS ONE*, *11*(3), e0150916. <https://doi.org/10.1371/journal.pone.0150916>
5. Hubbard DK, Parsons KM, Bythell JC, Walker ND. The effects of Hurrican Hugo on the reefs and associated environments of St. Croix, U. S. Virgin Islands- a preliminary assessment. Journal of Coastal Research 1992; 81(8), 33-48.
6. Japaud, A., Bouchon, C., Magalon, H., & Fauvelot, C. (2019). Geographic distances and ocean currents influence Caribbean Acropora palmata population connectivity in the Lesser Antilles. *Conservation Genetics*, *20*(3), 447–466. https://doi.org/10.1007/s10592-019-01145-9
7. Levitan, D. R., Fogarty, N. D., Jara, J., Lotterhos, K. E., & Knowlton, N. (2011). Genetic, spatial, and temporal components of precise spawning synchrony in reef building corals of the montastraea annularis species complex. *Evolution*, *65*(5), 1254–1270. https://doi.org/10.1111/j.1558-5646.2011.01235.x
8. Levitan, D. R., Fukami, H., Jara, J., Kline, D., McGovern, T. M., McGhee, K. E., Swanson, C. A., & Knowlton, N. (2004). Mechanisms of reproductive isolation among sympatric broadcast‐spawning corals of the montastraea annularis species complex. *Evolution*, *58*(2), 308–323. https://doi.org/10.1111/j.0014-3820.2004.tb01647.x
9. Monteiro, C. A., Serrão, E. A., & Pearson, G. A. (2012). Prezygotic Barriers to Hybridization in Marine Broadcast Spawners: Reproductive Timing and Mating System Variation. *PLOS ONE*, *7*(4), e35978. https://doi.org/10.1371/journal.pone.0035978
10. Ohki, S., Kowalski, R. K., Kitanobo, S., & Morita, M. (2015). Changes in spawning time led to the speciation of the broadcast spawning corals Acropora digitifera and the cryptic species Acropora sp. 1 with similar gamete recognition systems. *Coral Reefs*, *34*(4), 1189–1198. https://doi.org/10.1007/s00338-015-1337-4
11. Prata, K. E., Bongaerts, P., Dwyer, J. M., Ishida, H., Howitt, S. M., Hereward, J. P., Crandall, E. D., & Riginos, C. (2024). Some reef-building corals only disperse metres per generation. *Proceedings of the Royal Society B: Biological Sciences*, *291*(2027), 20231988. https://doi.org/10.1098/rspb.2023.1988
12. Rosser, N. L. (2015). Asynchronous spawning in sympatric populations of a hard coral reveals cryptic species and ancient genetic lineages. *Molecular Ecology*, *24*(19), 5006–5019. <https://doi.org/10.1111/mec.13372>
13. Smith TB, Brandtneris VW, Canals M, Brandt ME, Martens J, Brewer RS, Kadison E, Kammann M, Keller J, Holstein DM. Potential structuring forces on a shelf edge upper mesophotic coral ecosystem in the U.S. Virgin Islands. Frontiers in Marine Science 2016; 3. <https://doi.org/10.3389/fmars.2016.00115>
14. Stoffle, B., Stoltz, A., Crosson, S., & Tookes, J. S. (2020). In the Wake of Two Storms: An Impact Assessment of Hurricanes Irma and Maria on the St. Croix and St. Thomas Fisheries, USVI. *The Applied Anthropologist*, *40*.
15. Thompson, D. M., Kleypas, J., Castruccio, F., Curchitser, E. N., Pinsky, M. L., Jönsson, B., & Watson, J. R. (2018). Variability in oceanographic barriers to coral larval dispersal: Do currents shape biodiversity? *Progress in Oceanography*, *165*, 110–122. https://doi.org/10.1016/j.pocean.2018.05.007
16. Wood, S., Baums, I. B., Paris, C. B., Ridgwell, A., Kessler, W. S., & Hendy, E. J. (2016). El Niño and coral larval dispersal across the eastern Pacific marine barrier. *Nature Communications*, *7*(1), 12571. <https://doi.org/10.1038/ncomms12571>

All changes to in-text and full references are included in our revised manuscript.

**Reviewer Comments to Author:**

**Reviewer #1:**

Evaluating how population and community structure is influenced by environmental conditions is key to understanding the influence of environmental change on ecosystems and implementing effective restoration/conservation efforts. This study explores the genetic structure of coral communities around St. Croix and how that genetic structure relates to a spatiotemporal variation in environmental conditions. The authors find variable genetic structuring across coral taxa with certain environmental conditions of key influence on coral community structure over space and time.

This is my second review of this manuscript. The authors have made useful modifications and additions to all sections of the manuscript that assist in bolstering their approach. The revised version of this manuscript is much improved. Although the hypothesis outlined in the introduction is relatively broad, I feel that the updated context of the approach assists in the interpretation of the results. I have a few lingering comments that should be addressed and may benefit the manuscript further.

L89-100: In the previous version of the manuscript I noted that this paragraph would be best if placed in the methods section. Although the revised introduction adequately addresses my other comments I still feel that a description if the specific gradient forest methods here is out of place. Although these methods are certainly key to the analytical structure of the assessment, noting them in the introduction feels abrupt, especially after the paragraph noting the hypothesis examined but before the description of study area. Given that the gradient forest methods aren’t the key focus of this study, rather environmental drivers of cryptic coral composition, the introduction would benefit from a reduced focus on the particular methods.

**>>>Response –** We moved this paragraph to the Methods (now lines 256-267).

Supplementary Figures: These appear to be out of order in the main text. Although this will probably be addressed in copy-editing, it may be best to rearrange them at this stage.

**>>>Response –** We re-ordered all supplementary figures and tables to be consistent with their order in the main text.

Semivariograms and kriging approach:
I appreciate the authors providing these supplementary figures—they greatly enhance the interpretation of the spatial interpolation of environmental conditions. Although, it is unclear what the numbers next to each point represent. Also, shouldn’t the points be pairwise comparisons of environmental stations? If so, shouldn’t there be many more points for each plot which may improve model fit.

**>>>Response –** Data points are grouped into bins based on their lag distance, so that all pairs of points with similar distances are analyzed together. Thus, each point in the variogram plot represents the average semivariance for all data pairs within a bin. The label next to each point indicates the number of data pairs used to calculate the semi-variance at each distance bin.

We added this explanation along with the following text to clarify the variogram plots in the supplementary figures:

*Supplementary Figure 3: “Semi-variograms are included to the right of each map, which depict spatial autocorrelation between environmental measurements across the seascape. The x-axis of the variogram plot refers to the “lag distance,” or the distance between pairs of data points (expressed here in degrees of latitude and longitude). Data points are grouped into bins based on their lag distance, so that all pairs of points with similar distances are analyzed together. Thus, each point in the variogram plot represents the average semivariance for all data pairs within a bin. By default, the R package automap ensures that each bin contains at least five data pairs; if any bin has fewer than five pairs, it is merged with the adjacent bin. The y-axis represents the calculated semi-variance at each distance bin. Finally, the label next to each point indicates the number of data pairs used to calculate the semi-variance at each distance bin.*

*From the resulting variogram plots, several parameters controlling the fit of the model can be interpreted- including the “nugget,” or the y-intercept of the variogram, which represents small-scale variability of the data likely attributed to measurement error. The “range” is the distance where the variogram levels off (i.e., distance where spatial autocorrelation reduces), and the “sill” is the variance where the variogram levels off.*

*When interpreting the variogram, lag distance increases along the x-axis. For certain variables, such as mean temperature, the semivariance on the y-axis generally increases, indicating a reduction in spatial correlation between data points as they become further apart. For other variables, such as minimum temperature, semivariance appears relatively constant across lag distances, implying a more uniform spatial distribution of values through space.”*

As the authors note, the fit of these models is quite variable: some perform better than others. Based on even the good fits, I am somewhat concerned about the spatial scale of interpolation. For example, the fit for DO_max isn’t very good over the distance of ~0.03 (what are the units here?). For other variables (e.g., monthly range in Nitrogen), the fit line doesn’t appear to fit the [negative] trend at all. Given these fits, I am concerned that the environmental patterns over space may not be adequately represented by the models described. In general, some of the fits appear to either (1) obscure the spatial variability inherent to some variables or (2) don't appropriately capture trends.

**>>>Response –** We agree that the environmental patterns are a critical factor and have taken the following steps to address your concerns-

To improve the fit of each variogram, we re-interpolated the variables individually, allowing us to visually inspect and refine the model fit for each case rather than relying solely on an automated kriging procedure. This iterative approach enabled us to ensure better alignment between the variogram models and the observed data. The updated interpolated variables are presented in **new Supplementary Figure 3** (included in a separate PDF attachment to preserve figure resolution).

While we believe these adjustments have generally improved the overall fit of the models, we acknowledge that some variograms still exhibit relatively high variability, particularly at smaller lag distances. We provided additional explanation (see pasted lines below), justifying why we consider this variability acceptable for our analysis. Furthermore, we note that repeated testing of various model fits did not substantially alter the overall spatial patterns, suggesting that the models are adequately capturing the larger-scale environmental trends despite local inconsistencies.

*Supplementary Figure 3: “Regarding the fit of the variogram model, we note that the initial points in the plot often exhibit high variability due to the small lag distances between data pairs. These points are calculated from fewer data pairs, as the number of available pairs tends to increase with larger distances. Moreover, the high variability among the initial points is often an artifact of observations being sparse or clustered around certain regions.*

*For example, in our study, the high variability at small lag distances in all variables is most likely due to more frequent sampling near the capital, Christiansted (see Supplementary Fig. 1), where environmental monitoring efforts are more concentrated. These localized measurements contribute disproportionately to the small lag distance bins. To minimize the impact of this artifact, we fit the variogram model to the remaining data points, excluding the early, high-variability points.*

*Some variogram models show a better fit than others (ex. mean nitrogen, mean dissolved oxygen, and mean temperature), thus the resulting interpolations for those variables are more likely to capture actual trends across the seascape. On the other hand, models with a poorer fit can still identify regions with exceptionally high values (ex. mean E. coli) or low values (ex. monthly range of Nitrogen). However, we opted to retain even these gradients as predictors to see if those spatial patterns correlated with genetic structure. If variables with weaker variograms demonstrated high associations with genetic structure, we would have interpreted that result as genetic turnover across the seascape without attributing it to our interpolated variables, as even well-predicted variables would need to be validated experimentally. We did not, however, retain variables without variability across the seascape (ex. minimum nitrogen) and removed those variables as predictors from gradient forest models.”*

Regarding your concern about the units for DO, we added clarification for units of all variables in the manuscript:

*Lines 210-211: “Variables included pH, Enterococcus and E. coli (count per 100ml), dissolved oxygen, Kjeldahl nitrogen, and phosphorus (mg/L), and Secchi disk depth (m).”*

Following the re-interpolation of our environmental variables, we also redefined the ecoregions and re-ran the gradient forest model using the updated set of variables. The resulting ecoregion patterns were consistent with those derived from the previous variables, and we have **updated Figure 2** accordingly to reflect this.

In terms of model outcomes, after re-running the gradient forest analysis, we found that depth remained by far the most important variable, followed by pH_yearly_range as the second most influential factor, followed by temperature-related variables (see **new Figure 5**). It is worth noting that the importance of pH_yearly_range is consistent with our initial manuscript submission, where it was also identified as the second most important variable.

**Reviewer #2:**

1) Overall I thought the revised text was fairly superficial, but it more or less addresses the point. For example, the phrase “stepped clines driving tension zone formation and assortative mating” is quite jargony and “other factors, such as ocean currents influencing larval dispersal” is quite vague. The statement that “the ability of currents to sustain high dispersal and connectivity” (L462) seems at odds with all the research showing that larvae (including corals) disperse much less than expected from ocean currents. For example: Prata et al 2024 Some reef-building corals only disperse metres per generation. Proc. R. Soc. B 291: 20231988. <https://doi.org/10.1098/rspb.2023.1988>

**>>>Response –** We omitted these vague and jargony sentences, and we added the following new paragraphs to our discussion that consider alternative explanations for cryptic community structure. These paragraphs focus on possible non-environmental drivers that could have influenced the patterns we observed but were beyond the scope of our current analysis. Specifically, we discuss factors such as ocean currents, reproductive strategies (e.g., brooding vs. spawning), natural disturbances, and prezygotic barriers, including gamete incompatibility and temporal isolation due to differences in spawn timing.

Regarding your comment on ocean currents and larval dispersal, we acknowledge that Prata et al. 2024 highlights the limited dispersal distances of reef-building corals. We revised our discussion point to clarify that while ocean currents may influence connectivity, the actual dispersal distance for some species could be much shorter.

*Lines 456-511: “While we can estimate the extent of environmental differences that contribute to community structure, we recognize that most lineages are not strictly confined to specific depths or ecoregions and can co-occur at certain sites (see Fig 4). Untangling the mechanisms driving and maintaining genetic differentiation between cryptic lineages in the absence of geographic isolation remains a challenge. In addition to environmental variables, non-environmental factors such as ocean currents, reproductive strategies, natural disturbances, and prezygotic barriers may also influence coral community structure. For example, ocean currents can restrict dispersal patterns of local coral taxa (Fiesinger et al., 2023, Wood et al., 2016) and determine the settling locations of coral larvae (Thompson et al., 2018). Seasonal variation in the strength and direction of currents can further affect larval connectivity (Gilmour et al. 2016). In St. Croix, currents bend around the southern coast, sending wake flows to both the eastern and western sites (Chérubin & Garavelli, 2016), which may help explain the structured distribution of cryptic lineages across the east and west. Additionally, the reproductive mode of coral species- whether brooding or spawning- influences dispersal distance due to variations in larval phase length. For instance, the broadcast spawner Acropora palmata can have parent-offspring separations of 70 meters to one kilometer (Aurélien et al.), while brooding Agaricia corals typically only disperse 2 to 11 meters per generation (Prata et al. 2024). Despite the influence of ocean currents, many coral larvae settle in close proximity to their parent colonies (Prata et al. 2024), suggesting that short dispersal distances may limit the role of currents in driving genetic differentiation within these populations.*

*Natural disturbances, such as hurricanes, can also shape the structure of cryptic coral communities by fragmenting corals and redispersing them over large distances. However, the impact of these disturbances varies depending on oceanic geographic features. For example, patterns of reef destruction in St. Croix caused by Hurricane Hugo in 1989 varied by depth, shoreline orientation, and the composition of benthic communities before the storm (Hubbard et al. 1991). Moreover, patterns of coral growth on the mesophotic shelf edge of the U.S. Virgin Islands appear to be structured by acute but infrequent swell impacts, which varies across depths (Smith et al. 2016). These examples illustrate how natural disturbances can generate distinct patterns in coral community structure, shaped by the interaction between damage and recovery processes. On St. Croix, the impacts of Hurricanes Irma and Maria were similarly devastating, with significant damage to heritage and fisheries resources (Dunnavant et al. 2018; Stoffle et al. 2020), though the full scope of damage to the island’s coral communities remains unclear.*

*Prezygotic barriers, such as temporal isolation and gamete incompatibility, can also drive genetic divergence and the formation of cryptic lineages in corals. Within a single species, individuals may spawn at different times of the year, with some spawning in spring and others in fall. This asynchronous spawning is observed in species such as Acropora tenuis, A. samoensis, A. digitifera, Orbicella spp., and Mycedium elephantotus, all of which show genetic differentiation linked to their spawning periods (Dai et al., 2000; Gilmour et al., 2016; Levitan et al., 2011; Ohki et al., 2015; Rosser, 2015). Spawn timing is also determined by environmental cues and genetics, and even just a few hours difference in spawning can lead to sympatric speciation (Monteiro et al. 2012). Gamete incompatibility further contributes to this process. In some Orbicella spp., eggs can demonstrate conspecific sperm precedence (CSP), where they preferentially accept sperm from their own species. CSP may help gametes from broadcast spawners find each other in the water column, but it may also drive divergence in gamete compatibility (Fogarty et al. 2012). In the Caribbean, three recently diverged Orbicella spp.- O. franksi, O. faveolata, and O. annularis- show varying levels of gamete incompatibility, which may have played a role in their speciation (Levitan et al. 2004).*

*Despite these alternative explanations for the observed distribution patterns, depth consistently appears to be a significant driver of cryptic genetic differentiation in prior literature (Grupstra et al. 2024) and in our study. In St. Croix, depth can explain geographically proximate yet genetically divergent coral populations. This is particularly evident in O. faveolata, S. siderea, M. cavernosa, and P. astreoides, all of which exhibit high lineage diversity along the extensive depth gradient in Ecoregion C (see Fig. 2 and Fig. 4). However, further exploration is warranted to investigate potential hybrid zones between cryptic lineages present on both sides of this ecological barrier, and to examine non-environmental drivers such as those described above.”*

2) Thanks for the info. I noted that no changes were made to the manuscript here. The point of my comment was just to make some edits that could help the reader (not just the reviewer). I think at least a statement in the manuscript that summarizes these connections would help the reader.

“Generally, environmental variables were monitored across all coastlines of St. Croix, and very close to coral sampling sites. The only exception is that E. coli was not monitored near Cane Bay on the north shore.” So this would be good to mention this is the manuscript.

**>>>Response –** We added the above context to the manuscript:

*Line 212-214: “All variables were monitored across all coastlines of St. Croix, and very close to coral sampling sites (Supplementary Fig 2). The only exception is that E. coli was not monitored near Cane Bay on the north shore.”*

Furthermore, we agree that clarifying the lineage connections would be beneficial for the reader. To help the reader trace lineages from St. Croix to lineages found in other regions, we have the following paragraph:

*Line 297-311: “When clustering A. agaricites and P. astreoides with samples from a prior study in Florida (Black et al., 2022), we observed that the two A. agaricites lineages from St. Croix cluster with two genetic lineages found throughout the Florida Keys. Specifically, the red St. Croix lineage (Fig 3a) clusters with the shallow-preferred lineage in Florida and the blue St. Croix lineage clusters with the depth-generalist lineage in Florida. However, the two P. astreoides lineages from St. Croix do not cluster with any of the lineages observed in the Florida Keys, and instead appear to be genetically differentiated. The M. cavernosa and S. siderea sample sets also clustered with samples from prior studies (Gallery et al., 2023; Rippe et al., 2021; Sturm et al., 2023). M. cavernosa from St. Croix clustered with six lineages specialized to various depths, and S. siderea clustered with two shallow and one deep-specialized lineage found across the Florida Keys and the Gulf of Mexico. Differentiation of three S. siderea lineages is apparent in hierarchical clustering and PCoA (Fig 3f), but the delineation of six M. cavernosa lineages is less obvious, especially in ordination space (Fig 3b). However, this is likely due to the under-sampling of M. cavernosa around St. Croix, and a larger sample set would likely reveal more striking differentiation of lineages.”*
